# Sustainable Polymer Composites for Thermal Insulation in Automotive Applications: A Systematic Literature Review

**DOI:** 10.3390/polym17162200

**Published:** 2025-08-12

**Authors:** Dan Dobrotă, Gabriela-Andreea Sava, Andreea-Mihaela Bărbușiu, Gabriel Tiberiu Dobrescu

**Affiliations:** 1 Faculty of Engineering, Lucian Blaga University of Sibiu, 550024 Sibiu, Romania; andreea.sava@ulbsibiu.ro (G.-A.S.); andreea.barbusiu@ulbsibiu.ro (A.-M.B.); 2 Academy of Romanian Scientists, Ilfov Street 3, 050045 Bucharest, Romania; 3 Department of Robotics and Manufacturing Systems, Faculty of Industrial Engineering and Robotics, National University of Science and Technology POLITEHNICA Bucharest, 060042 Bucharest, Romania; tibidobrescu@yahoo.com

**Keywords:** industrial applications, polymers, composite materials, eco-design, thermal insulation

## Abstract

This systematic literature review explores recent advancements in polymer-based composite materials designed for thermal insulation in automotive applications, with a particular focus on sustainability, performance optimization, and scalability. The methodology follows PRISMA 2020 guidelines and includes a comprehensive bibliometric and thematic analysis of 229 peer-reviewed articles published over the past 15 years across major databases (Scopus, Web of Science, ScienceDirect, MDPI). The findings are structured around four central research questions addressing (1) the functional role of insulation in automotive systems; (2) criteria for selecting suitable polymer systems; (3) optimization strategies involving nanostructuring, self-healing, and additive manufacturing; and (4) future research directions involving smart polymers, bioinspired architectures, and AI-driven design. Results show that epoxy resins, polyurethane, silicones, and polymeric foams offer distinct advantages depending on the specific application, yet each presents trade-offs between thermal resistance, recyclability, processing complexity, and ecological impact. Comparative evaluation tables and bibliometric mapping (VOSviewer) reveal an emerging research trend toward hybrid systems that combine bio-based matrices with functional nanofillers. The study concludes that no single material system is universally optimal, but rather that tailored solutions integrating performance, sustainability, and cost-effectiveness are essential for next-generation automotive thermal insulation.

## 1. Introduction

Thermal management is a key challenge in the transition toward energy-efficient and electrified vehicles. Conventional insulation systems are increasingly inadequate for meeting emerging demands in automotive design. Thermal insulation represents a critical challenge in automotive design, especially in the context of electrification, downsizing, and stringent environmental regulations. In this review, we examine how polymer composites contribute to thermal management in automotive components, bridging material science with functional engineering. Despite the rapid development of thermal insulation technologies, the current literature lacks an integrated analysis of how polymer composites respond to the unique thermal, mechanical, and sustainability requirements of the automotive sector. Fragmented findings, methodological variability, and limited cross-sectoral synthesis hinder the ability to identify optimal material solutions. Therefore, this review addresses a critical gap by systematically evaluating polymer composite systems, manufacturing techniques, and insulation strategies aligned with automotive applications. Given the increasing demand for lightweight, thermally efficient, and sustainable automotive components, the optimization of polymer composites for insulation purposes represents a critical research priority. Nevertheless, a comprehensive synthesis that connects material performance, recyclability, and automotive-specific constraints remains absent from the current literature. This review addresses that gap by offering a systematic and comparative analysis.

Currently, composites, including polymer resins, play an essential role in modern production due to technological developments that allow the innovative integration of various materials and chemical compounds [1]. The chemistry of these resins is particularly interesting, attracting the attention of many researchers [2,3] who emphasize advances in material properties and innovative techniques that improve their efficiency and reliability. Their contributions highlight the impact of these materials on enhancing electrical insulation, providing solutions to challenges encountered in high-performance applications.

The production of polymer resins often involves the use of fossil energy sources, such as petroleum and natural gas. Their processing in refineries and petrochemical plants significantly contributes to environmental pollution [4]. Resins are viscous compounds that, through the curing process, transform into rigid polymers, containing reactive functional groups such as acrylates or epoxides [5].

They also represent a special class of highly reactive prepolymers, containing epoxy groups in their molecular structure, where the term “epoxy” refers to the final cured product of an epoxy resin [6,7]. In recent years, the use of high-performance polymer resins in the industrial components industry has significantly increased due to their superior properties, such as mechanical strength, chemical stability, and thermal insulation [8].

These types of resins are widely used across various industries, from adhesives and microelectronics to matrices for advanced fiber-reinforced composites, where materials with high thermal conductivity are essential in fields such as the electronics and aerospace industries [9,10]. The processing of epoxy resins is beneficial due to their ability to create compositions with adjustable rheological properties, including low viscosity, and due to the diversity of available curing agents. This allows curing at both ambient and high temperatures [11]. Curing agents play a crucial role in the formulation of aqueous epoxy resin systems, but their application is limited by the low efficiency of the emulsification process [12]. Additionally, the mixing ratio, curing time, and temperature influence the microstructure and final performance of the epoxy resin [13].

Composite materials are composed of two or more distinct components, each with its own characteristics, resulting in a material with enhanced properties that also possesses a high degree of compatibility, such as mechanical strength, thermal conductivity, or electrical conductivity [14,15]. Although epoxy resins have great adaptability and superior performance, they can be optimized by adding additives such as graphene, carbon nanotubes, silicon carbide, rubber particles, or polyhedral oligomeric silsesquioxane (POSS) [16].

The addition of rigid groups to the composition of epoxy resins is one of the main methods for enhancing thermal conductivity [17]. Recent research analyzes the relationship between the flexibility of the monomeric chains in epoxy resins and the thermal conductivity of modified epoxy resins [17].

Yet, resin-based materials present notable limitations, including low resistance to high temperatures, reduced thermal conductivity, and weaker mechanical properties, which restrict their use in demanding environments. To improve these characteristics, researchers have developed composites with an insulating resin matrix, enriched with fillers or fibers to enhance thermal conductivity [3].

Despite their advantageous properties, epoxy resin-based materials exhibit susceptibility to thermal degradation under high-temperature conditions [18]. This phenomenon depends on the type of polymer and the aging conditions analyzed [18]. Currently, natural resins have been almost entirely replaced by synthetic resins, which are divided into two categories: heat-resistant and thermoplastic resins [19,20].

In an era of sustainable development, there is a growing need for the development of durable and recyclable composite materials that contribute to reducing carbon emissions and protecting the environment. Recycling polymer matrix composite materials continues to pose a major challenge due to their complex structure and thermoset nature [21]. During the synthesis process of epoxy resins, the use of anhydride-based curing agents and tertiary amine accelerators leads to the formation of rigid polymers with complex structures. This makes the recycling process more difficult and increases the negative environmental impact [22].

Recently, researchers [23] have developed methods for pre-treating epoxy resins before degradation, thus highlighting the importance of this stage in the recycling process.

Epoxy resins are a class of high-performance thermosetting polymers known for their excellent mechanical strength, chemical resistance, dimensional stability, and strong adhesion to various substrates. Their low viscosity, tunable curing behavior, and compatibility with different fillers make them ideal for producing advanced composite materials used in structural and insulating applications. These characteristics have led to their extensive use across the automotive, aerospace, and electronic sectors.

Despite these advantages, epoxy resins pose significant environmental challenges. Their synthesis typically involves fossil-derived precursors and complex crosslinked networks that hinder recyclability. Moreover, their degradation at elevated temperatures can generate toxic byproducts, and their disposal at end-of-life stages raises ecological concerns.

In the automotive sector, thermal insulation plays a vital role in optimizing vehicle energy efficiency, ensuring passenger comfort, and protecting sensitive electronic components from overheating. Polymer composites are increasingly used in under-the-hood insulation panels, battery enclosures, and HVAC systems due to their low thermal conductivity, lightweight properties, and formability. Moreover, with the rise of electric vehicles (EVs), effective thermal management has become a critical design parameter. For instance, thermal runaway prevention in lithium-ion battery systems relies heavily on insulating materials with precise thermal resistance and fire-retardant capabilities. In this context, the integration of polymer composites into thermal insulation solutions offers a path forward for balancing performance, weight reduction, and sustainability in modern automotive engineering. Therefore, this review aims to explore and systematize current knowledge on the role of sustainable polymer composites in advancing thermal insulation technologies specifically tailored for automotive applications.

Considering all the aspects mentioned above, this article aims to provide a detailed analysis of the properties, uses, and challenges associated with polymer resins.

## 2. Research Questions Regarding Insulation Materials

Although the concept of component insulation has been studied and applied over time, there are still numerous debates and challenges related to

- The role of insulation in components and its influence on the strength and performance of systems.
- Identifying suitable materials for insulation, considering both technical performance and sustainability.
- Approaches through which insulation materials can be optimized to provide superior performance while reducing costs and ecological impact.

Furthermore, as performance requirements become increasingly stringent and sustainability becomes a global concern, a reevaluation of current insulation solutions is required. It can be anticipated that technological advancements in the field will lead to more efficient materials that will enhance both the reliability of components and the energy efficiency of products. Table 1 presents the main materials from the polymer family identified, along with their advantages and disadvantages.

Based on the challenges identified earlier, the aim of this paper is to analyze and clarify the key aspects of component insulation. Furthermore, strong arguments will be presented to demonstrate the hypothesis that advancing insulation materials can have positive effects on costs, the environment, and technological development. Thus, the article will thoroughly examine the questions “why,” “which,” “how,” and “what” in the context of component insulation.

Q1—**Why** is component insulation important, and how does it influence the durability and efficiency of products?

Q2—**Which** materials are most suitable for component insulation, and what criteria should be considered in this selection?

Q3—**How** can insulation materials be optimized to enhance performance and sustainability?

Q4—**What** future research is needed to improve the performance of insulation materials and develop innovative solutions?

Based on the challenges identified in the literature, this review focuses on four core thematic directions that structure the subsequent analysis. First, it explores the role and importance of component insulation in enhancing product durability and operational efficiency. Second, it examines the most suitable polymer-based materials for insulation, considering both technical performance and sustainability criteria. Third, it analyzes strategies for optimizing these materials to improve thermal performance while reducing ecological impact and cost. Finally, the review identifies future research directions aimed at addressing current limitations and promoting innovative, sustainable solutions for thermal insulation in automotive applications.

## 3. Literature Review Methodology

### 3.1. Search Strategy and Selection Criteria

**Figure 1:**
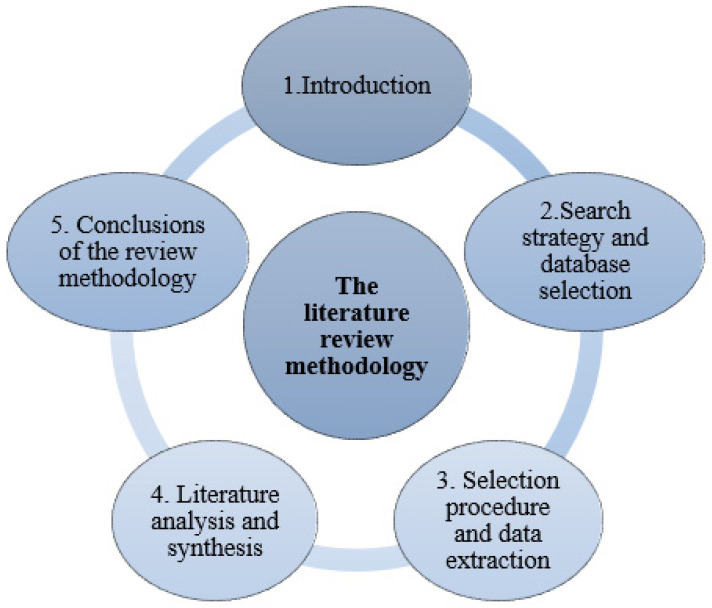
Steps used for analysis studied.

The answers to the questions formulated above, along with the formulation of hypotheses, were generated through a systematic approach based on a detailed analysis of databases from the existing scientific literature. These conclusions were based on an analysis of information extracted from specialized scientific articles, followed by a thorough classification of these sources according to their theme and relevance. The process also included a critical evaluation of the results, considering the research context, to ensure a solid foundation for the hypotheses and conclusions drawn. As illustrated in Figure 1, the literature review followed a structured sequence of steps, beginning with the formulation of research questions, followed by the identification of relevant databases and search terms, screening and eligibility assessment of the retrieved records, and culminating in the extraction and synthesis of key data.

The literature review represents an important step in any research process, as it allows for the identification of gaps, highlights trends, and establishes a solid theoretical foundation. In the context of studying the mechanisms of aging and degradation of electrical insulating materials, the methodological approach should outline how various factors interact to produce microstructural changes that influence the performance of insulators.

To ensure comprehensive coverage of the scientific literature, the following databases were accessed:

- MDPI
- ScienceDirect (Elsevier)
- Scopus
- Web of Science
- Wiley
- SpringerLink

These sources provide access to indexed articles and adhere to international scientific standards.

Keywords were chosen based on the objectives of the study and included terms such as electrical insulating materials, polymeric material degradation, composite materials, microstructural modifications, thermal stress, polymer recycling, and process insulation. Filters were applied based on publication dates (e.g., the last 10–15 years, with some exceptions for fundamental sources) and language (English, except where significant contributions were found in other languages).

**Figure 2:**
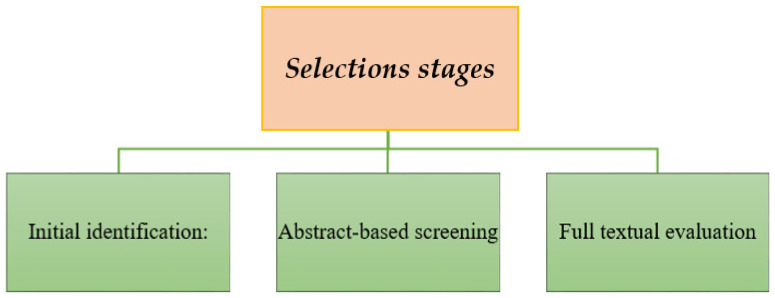
Literature Selection Stages in the Systematic Review.

As shown in Figure 2, the literature selection process was divided into three main stages: initial identification, abstract-based screening, and full-text evaluation. This systematic approach ensured that only studies directly addressing environmental influences, microstructural changes, or degradation mechanisms were retained for analysis. The figure provides a concise visual overview of the inclusion and exclusion flow applied throughout the review process.

- Initial identification—Extensive searches were conducted in the mentioned databases using the established keywords and filters. In this stage, a significant number of articles were collected, which were later reviewed based on their titles and abstracts.
- Screening based on abstract—Each article was evaluated for relevance based on the abstract. Studies that did not directly address the impact of environmental factors, microstructural changes, or aspects related to degradation identification were excluded.
- Full-text evaluation—The articles selected in the screening phase were read in full to verify whether they met the necessary criteria.

**Figure 3:**
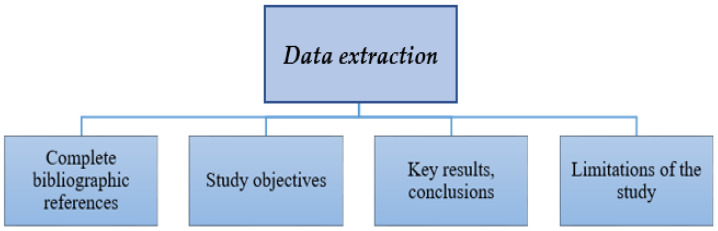
Key Data Extraction Categories.

Figure 3 presents key categories of data extracted from selected studies: bibliographic information, study objectives, key findings, limitations, and future research directions.

For each final study included, the following information was recorded:

References complete bibliography: authors, title, year, journal, volume, pages, and DOI.

Objectives studied and research questions addressed.

Results and key conclusions regarding the relationship between changes at the microstructural level and performance materials.

### 3.2. Review Protocol

After collecting the relevant sources, these were examined through a comparative approach, considering the following:

- Thematic classification—The sources were grouped according to the main topics identified: properties of polymer materials, optimization methods for component insulation, recyclability, and sustainability.
- Identification of research gaps—The focus was on highlighting issues that were insufficiently analyzed, which led to the formulation of directions for future research.
- Time period—The search for articles was limited to those published in the last 10–15 years, with some exceptions for basic sources to obtain updated information. This approach may exclude fundamentally older studies, which, although valuable, may not reflect the most recent technological evolution.
- Language—The focus was on articles in English (and occasionally Romanian), which may have led to the exclusion of some helpful works published in other languages.
- Databases—Although key recognized databases were accessed, there is a possibility of omitting studies from lesser-known sources.

**Figure 4:**
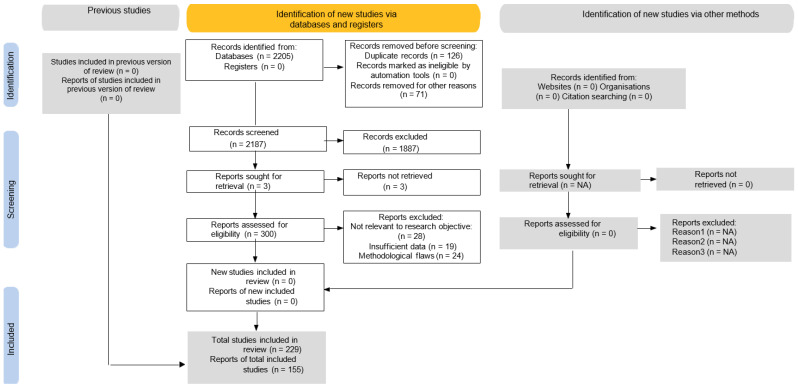
PRISMA study selection flow chart.

To ensure methodological transparency and adherence to international standards for systematic reviews, a PRISMA flow chart was employed. The PRISMA flow diagram (Figure 4) was revised to explicitly state the total number of records identified (n = 2205), duplicates removed (n = 126), titles and abstracts screened (n = 2187), exclusions based on criteria (n = 1887), and full texts assessed and included (n = 229). The PRISMA flow diagram is presented in Figure 4. A completed PRISMA 2020 checklist is provided in the Supplementary Materials.

### 3.3. Bibliometric Analysis and Keyword Mapping

To complement the systematic synthesis, a bibliometric analysis was conducted using VOSviewer (version 1.6.19), based on the final selection of 229 peer-reviewed articles retained after full-text screening. This analysis aimed to visualize the conceptual structure of the literature on sustainable polymer composites for thermal insulation in automotive applications.

**Figure 5:**
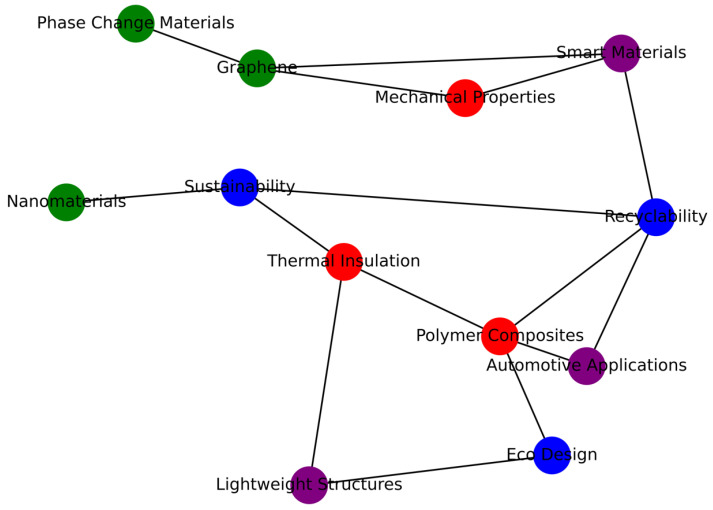
Keyword Co-Occurrence Map (VOSviewer-style).

The keyword co-occurrence network (Figure 5) was generated by setting a minimum threshold of five keyword occurrences per term. Out of more than 1000 unique keywords, 53 met this threshold and were included in the map. The network visualization reveals several distinct clusters:

- The red cluster centers around the terms “*polymer composites*”, “*thermal insulation*”, and “*mechanical properties*”, reflecting a core research focus on material performance and characterization.
- The blue cluster includes keywords such as “*recyclability*”, “*eco-design*”, and “*sustainability*”, indicating a growing interest in environmentally responsible material development.
- The green cluster highlights terms like “*nanomaterials*”, “*phase-change materials (PCM)*”, and “*graphene*”, associated with advanced functionalization strategies for enhancing insulation behavior.

**Figure 6:**
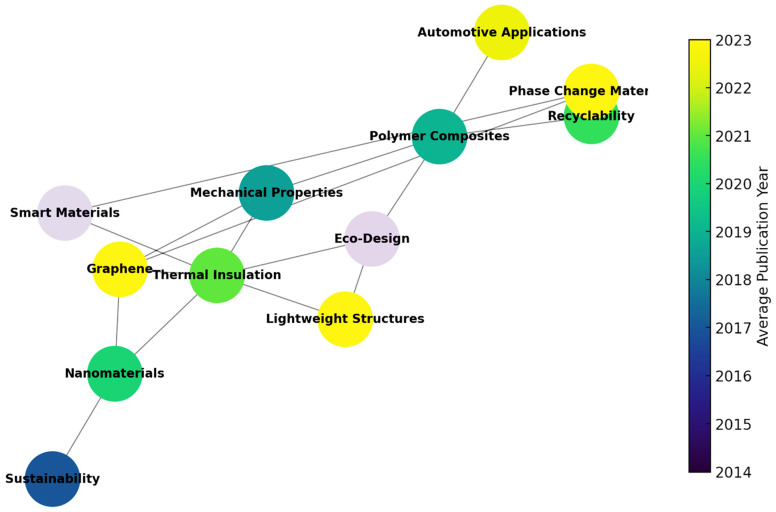
Overlay Visualization of Keywords by Average Year of Appearance.

These clusters demonstrate the interdisciplinary nature of the field, combining polymer science, thermal management, environmental engineering, and materials optimization. In addition, the overlay visualization (Figure 6) offers insight into the temporal evolution of topics. Figure 6 synthesizes the integration of these future directions into a cohesive development pathway for sustainable thermal insulation in automotive applications.

Early research (2010–2015) focused primarily on *polymer synthesis* and *insulation efficiency*. More recent studies (post-2018) emphasize *circular economy*, *bio-based materials*, *nanostructured composites*, and *automotive integration*. The emergence of keywords such as “*electric vehicles*”, “*lightweight structures*”, and “*smart materials*” suggests a shift toward application-driven and sustainability-oriented approaches. This bibliometric analysis reinforces the relevance of the thematic directions adopted in this review. It also confirms the presence of research gaps, particularly in the direct integration of sustainable polymer composites into automotive systems.

The literature review methodology enabled a detailed analysis of the available information, contributing to the foundation of the research hypotheses. By employing a systematic approach, this work provides a clear perspective on the innovations and challenges in the field of polymeric insulation materials, identifying future research directions and sustainable solutions for improving the performance of these materials.

To provide a structured overview of recent contributions, a synthesis table was developed summarizing key studies in the field. Table 2 of 20 selected references includes details on authorship, study aims, material systems, key findings, and specific application domains. This table presents a selected subset of reviewed articles for illustrative purposes.

## 4. Evaluation of Questions Q1, Q2, and Q3 Regarding the Insulation of Components with Polymeric Resins

**Q1—Why is the insulation of components important, and how does it influence the durability and efficiency of products?**

The insulation of industrial components plays an important role in maintaining their structural and functional integrity over the long term. Several factors, such as temperature fluctuations, humidity, exposure to chemicals, and intense mechanical stress, can lead to rapid material degradation, resulting in premature failures and significant economic losses [24].

Another key aspect is protection against corrosion and wear. For example, research conducted by [25] shows that applying epoxy resins to metal surfaces reduces oxidation by over 60%, which improves mechanical strength and decreases maintenance frequency. In addition, thermal insulation helps reduce energy losses. Another study conducted by [8] presented in the work “Materials Science and Engineering: An Introduction” demonstrated that the use of insulating polymer composites in industrial equipment can reduce energy consumption by 25% by maintaining an optimal operating temperature. Insulating materials can also prevent short circuits and equipment overheating, thus reducing the risk of fire. According to a report published by [26] in the work “Plastics: Materials and Processing (3rd Edition)”, flame-retardant polyurethanes used in the insulation of electrical components reduce the risk of fire by 35% compared to conventional materials.

Product lifetime and its extension have gained attention in the past two decades, particularly in the evolving fields of design for durability [27]. This study, “Composite Materials from Renewable Resources as Sustainable Corrosion Protection Coatings”, explores the use of epoxy resin and lignin-based composites for corrosion protection of metals. It was demonstrated that these composites offer effective corrosion protection due to the outstanding mechanical properties and chemical stability of epoxy resins [28].

**Figure 7:**
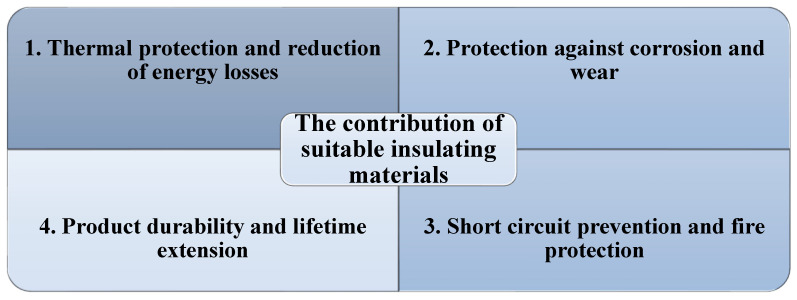
Key contributions of appropriate insulating materials to industrial component performance: thermal protection, energy loss reduction, increased service life, and lower maintenance costs.

The benefits of using appropriate insulating materials extend beyond physical protection and directly impact the energy efficiency and service life of industrial components. Figure 7 summarizes the key contributions of insulation to component performance, including thermal protection, maintenance cost reduction, and product longevity. This visualization reinforces the essential role of insulation in enhancing both technical and economic outcomes across industries.

Because the insulation of industrial components is an essential step for ensuring the durability and efficiency of industrial products, having a direct impact on extending the lifespan of equipment, reducing maintenance costs, and optimizing energy consumption, it serves multiple functions, including protection against thermal variations, humidity, corrosion, and mechanical stress. These factors can severely affect the long-term performance and reliability of industrial equipment.

- Thermal protection and energy loss reduction—Thermal insulation is a key element in protecting industrial components, helping maintain an optimal operating temperature and preventing energy losses. In industries like energy and chemicals, where controlled temperature is crucial for manufacturing processes, thermal insulation can significantly reduce operational costs by minimizing heat loss. Researchers [29] investigated the total energy consumption of a single-screw extruder, identifying optimization methods. The study “Monitoring and Modelling of the Energy Consumption in Polymer Extrusion” involved measuring energy consumption under different processing conditions and modeling it based on process variables. Results showed that the equipment’s energy demand is strongly influenced by machine, material, and process parameters. The proposed models demonstrated excellent agreement with experimental measurements and proved useful for optimizing energy efficiency in the process.
- Protection against corrosion and wear—Metal corrosion, caused by exposure to humidity, chemicals, and extreme temperatures, can significantly shorten the lifespan of industrial components. Adequate insulation can effectively mitigate this phenomenon by safeguarding sensitive materials and lowering both maintenance and replacement costs [25]. It was observed that the use of epoxy resins as an insulating material on metal surfaces can reduce oxidation by over 60%, improving mechanical strength and reducing maintenance frequency. Additionally, research by [28] highlighted that using composites based on epoxy resins and lignin (a complex organic polymer) can provide long-term protection against metal corrosion, due to their chemical stability and mechanical strength. Therefore, by implementing optimal insulation solutions, industrial equipment can endure longer in aggressive environmental conditions, contributing to maintaining its performance and reducing operational costs.
- **Prevention of short circuits and fire protection—**The insulation of electrical and electronic components is especially important for preventing short circuits and fires in industrial installations. Insulating materials not only protect electrical equipment from negative external factors but also prevent the risks of overheating. According to a report by [26] in the paper “Plastics: Materials and Processing (3rd Edition),” the use of flame-retardant polyurethanes in electrical component insulation can reduce the fire risk by 35% compared to conventional materials. These materials are essential for operational safety and for protecting industrial equipment and personnel.
- **Product durability and lifetime extension—**The long-term properties of polymers and their degradation processes are not opposing aspects. In fact, they are intrinsically connected through the balance between durability and degradation capacity [30]. Researchers [31] conducted a detailed analysis of the synthesis and degradation mechanisms of biodegradable polymers in natural environments, focusing on polymers such as PLA (polylactic acid), starch-based polymers, and vegetable fibers. They studied the interaction between abiotic and enzymatic factors in degradation processes, as well as the effects of chemical and physical changes on the stability and performance of these polymers.

The paper “Review of the Synthesis and Degradation Mechanisms of Some Biodegradable Polymers in Natural Environments” highlighted that polymer degradation is significantly influenced by environmental conditions such as temperature and humidity, as well as the presence of additives or treatments, such as nanocomposites and crosslinking. It was also shown that these interventions can be used to optimize the durability and extend the lifespan of biodegradable polymer-based products, ensuring environmental compatibility. This article provides valuable insights into improving biodegradable polymers to meet current environmental challenges [31].

In conclusion, the insulation of industrial components is important for ensuring their durability and efficiency over time. Protection against corrosion, wear, and energy losses and extending the lifespan of industrial components are essential elements affecting the efficiency and maintenance costs of equipment. Investments in innovative insulation solutions, such as thermoresistant materials, composites for corrosion protection, and flame-retardant materials, bring benefits not only in protecting industrial equipment but also in supporting a sustainable and energy-efficient future.

**Q2—What are the most suitable materials for insulating components, and what criteria should be considered in their selection?**

When choosing the most suitable materials for insulation, specific requirements for each industrial process must be taken into account. Some of the most commonly used materials for insulating components are presented in Table 3.

**Selection criteria**:

1. Type of insulation required (thermal, electrical, etc.).
2. Operating temperature and thermal resistance.
3. Mechanical properties—shock resistance, flexibility.
4. Chemical compatibility—resistance to aggressive substances.
5. Cost and availability.
6. Durability and resistance to aging.
7. Weight—important for mobile/aerospace applications.
8. Sustainability—ecological impact, recyclability.

Researchers [37], after conducting several analyses, concluded that polymeric foams are a very promising material for thermal insulation due to their excellent performance and versatility, playing a crucial role in the development of sustainable technologies. They help reduce energy losses and improve thermal efficiency in various fields. In the paper “A review of the state-of-the-art on thermal insulation performance of polymeric foams,” the following conclusions were made:

Theoretical modeling precision: The developed model for predicting the thermal conductivity of polymeric foams has proven to be accurate and can be used for the optimization of their design. It takes into account factors such as foam density and cell size.

Polymeric foam performance: These materials have been confirmed as among the most efficient for thermal insulation applications, with the potential to improve energy sustainability.

Practical perspectives: Polymeric foams are ideal for industrial applications due to their ability to combine excellent insulation properties with ease of processing and relatively low costs [37].

In conclusion, the selection of materials for isolating different industrial components depends on the specific requirements of the industrial process and the characteristics of each material, such as thermal, mechanical, and chemical resistance, durability, and ecological impact. Materials like epoxy resins, polyurethane, PEEK, silicones, and fiberglass composites are suitable for different applications. Polymeric foams, in particular, offer excellent thermal insulation performance and contribute to energy efficiency and sustainability in industrial processes.

**Q3. How can insulation materials be optimized to increase performance and sustainability?**

Recent research focuses on new methods for improving the performance and sustainability of insulation materials. These methods include adding nanomaterials, using eco-friendly polymers, advanced manufacturing technologies, and integrating hybrid materials. Furthermore, emerging technologies, such as self-healing and nanostructuring, play a key role in extending durability and enhancing insulation performance.

- **Nanomaterials for improving thermal and mechanical performance**

The integration of nanomaterials like carbon nanotubes (CNT), graphene, and zinc oxide has shown significant improvements in insulation material properties. Carbon nanotubes are used for their low thermal conductivity and high mechanical strength to reinforce polymer matrices [38], concluding that these composite materials can be applied in various industries, such as construction, automotive, and electronics, where thermal control is essential for product performance and safety.

Researchers [39] have demonstrated that graphene can serve as a flexible filler material, leading to the development of nanocomposites with various energy applications. They also emphasized the importance of graphene in improving the performance of polymeric materials and opening new opportunities for energy applications. Additionally, it was analyzed how graphene, due to its very high thermal conductivity, can be used as a filler material to enhance the thermal conductivity of polymer composites. In the paper “Thermal Conductivity of Graphene-Polymer Composites: Mechanisms, Properties, and Applications,” researchers demonstrated that graphene can significantly improve the thermal performance of polymer composites, opening up new opportunities for their use in thermal management for electronic and optoelectronic devices [40].

- **Recyclable and biodegradable materials to reduce the carbon footprint**

The use of eco-friendly and biodegradable materials represents a significant research focus to reduce the ecological impact of insulation materials. Researchers at ZymoChem have demonstrated that their biotechnology process uses microbes to transform renewable sources into sustainable alternatives to petroleum-based polymers. This method offers a more accessible process and more efficient production compared to other sustainable material development solutions [41].

Biodegradable polymers, particularly aliphatic hydroxyacid polyesters, can replace synthetically produced polymers that are difficult to decompose biologically due to their similar properties. These can be produced from renewable resources and processed with equipment used for polyolefins or other synthetic plastic materials. The mechanical properties of PHA vary depending on the monomer structures and the molecular weight of the polymers, allowing the production of biodegradable polymers with a wide range of applications, including packaging, food additives, biomedical materials, and biosensors [42].

In the article “Green synthesis of hypercrosslinked polymers for CO_2_ capture and conversion: recent advances, opportunities, and challenges,” the ecological synthesis of hypercrosslinked polymers for CO_2_ capture and conversion was explored, where researchers demonstrated that hypercrosslinked polymers are promising systems for CO_2_ capture and conversion due to their large specific surface areas, variable pore characteristics, superior stabilities, and modifiable surface functionalities. Their analysis for [43] highlighted green synthesis methods for HCPs and emphasized their use in CO_2_ fixation and separation, with a focus on selectivity and CO_2_ recoverability. The potential of HCPs in CO_2_ conversion into valuable compounds was also explored for [43].

Ref. [44] validated existing experimental data through a proposed multiscale model, suggesting that this approach can be effectively used for the design of polymer composites reinforced with grafted nanoparticles.

- **Advanced manufacturing technologies: 3D printing and controlled polymerization**

The term “3D printing” refers to various methods in which material is deposited, connected, or solidified under computer control to form a 3D article, with the material being placed layer by layer, such as polymers, concrete, plastics, liquids, or powder granules [45].

Several new methods, including 3D printing of polymers with nickel electroforming, have been tested to create complex metal structures, such as electrodes for streamer discharge plasma generators. The study “Hybridizing 3D printing and electroplating for the controlled forming of metal structures within fused deposition modelled contours” demonstrated that this hybrid approach allows selective operation of substrates and offers an affordable alternative for manufacturing large and detailed metal components [46].

Researchers [47] have conducted tests to control molecular weight and its distribution, achieving over 90% monomer conversions in minutes. They also tested the mechanical properties of printed objects by adjusting the resin components to vary the material characteristics. This study demonstrated the potential of these technologies in creating customized materials for various fields [47].

Through various tests, photopolymerizable resins with improved mechanical and functional properties were developed, as well as for integrating smart materials like piezoelectric ceramics into DLP processes. Additionally, the use of artificial intelligence for optimizing processes and recycling DLP-type resins has been explored. Researchers [48] highlighted the capacity of these technologies to support sustainable manufacturing practices and extend the applications of advanced materials [48].

Hybrid materials that combine organic and inorganic components have also been considered, offering innovative solutions for thermal and chemical protection. The studies by researchers [49] explore enzyme stability in hybrid polymer-based nanogels containing phosphorylcholine groups. Through these analyses, copolymer nanogels were synthesized via RAFT polymerization, and the enzyme β-galactosidase (β-gal) was immobilized within these nanogels. The study entitled “Enzyme stability in polymer hydrogel–enzyme hybrid nanocarrier containing phosphorylcholine group††Electronic supplementary information (ESI) available” demonstrated that these hybrid nanogels offer superior enzyme stability compared to free enzymes, protecting them from inactivation caused by organic solvents, proteolytic enzymes, and elevated temperatures [49]. A significant focus is placed on molecular self-assembly and innovative strategies for constructing three-dimensional HOI materials.

These materials are evaluated for their protective characteristics and structural reliability in corrosive or flammable environments. Their applications include catalysis, energy storage devices, and biomedical uses, due to the functional properties imparted by their architecture and composition [50].

Another suitable material is the multifunctional hybrid aerogel, integrating organic-inorganic-metal components, designed for excellent thermal insulation and protection against electromagnetic interference. Researchers [51] have demonstrated that the ternary hybrid aerogel based on boron-silicon-tantalum is a high-efficiency multifunctional alternative for safety in extreme conditions. They showed that this material offers excellent thermal performance, superior mechanical strength and thermal stability, and remarkable electromagnetic shielding properties and also highlighted its potential for industrial and aerospace applications due to its ability to combine thermal protection, mechanical resistance, and electromagnetic shielding [51].

- **Self-healing techniques for extending insulation lifespan**

Self-healing technologies are employed to extend the service life of insulation materials and reduce the need for maintenance or repair. The latest advances in self-healing polymers with various strategies have been explored, such as:

- Reversible covalent bonds: Materials can heal by re-establishing chemical bonds.
- Dynamic physical bonds: Molecular interactions that enable healing without compromising mechanical properties.
- Microcapsules and channels: Systems that release healing agents in damaged areas.

Perspectives on the use of bio-based monomers and the contribution of these materials to the circular economy are also discussed [52]. By analyzing two main types of self-healing materials—extrinsic ones that require capsules or vascular networks to release healing agents, and intrinsic ones that rely on molecular interactions or reversible bonds—it has been demonstrated that these materials can restore mechanical properties, offer corrosion protection, increase electronics durability, and contribute to energy storage. Although challenges such as high production costs and synthesis complexity persist, these materials demonstrate significant potential to transform a wide range of industries [53].

Researchers [54] have explored various types of dynamic bonds used in these materials, namely:

- Diels–Alder reaction—This type of reaction allows the reversible reformation of covalent bonds, enabling repeatable self-healing capabilities.
- Metallic bonds—These provide a combination of good mechanical properties and healing ability.
- Hydrogen and ionic bonds—These non-covalent interactions allow healing at low temperatures and under mild conditions.
- Disulfide bonds—These are used to create self-healing materials in oxidative environments.

Ref. [54] demonstrated that these materials can repair scratches, cracks, and other mechanical damage, thereby extending the service life of materials. The study highlights current issues such as balancing repair efficiency with mechanical properties and offers prospects for the future development of polymers with high self-healing capacities.

- **Nanostructuring of materials for enhanced performance**

The methods for processing nanostructured polymers and advanced nanocomposites have been thoroughly analyzed by [55], highlighting the importance of nanostructure control to achieve improved material properties.

**Figure 8:**
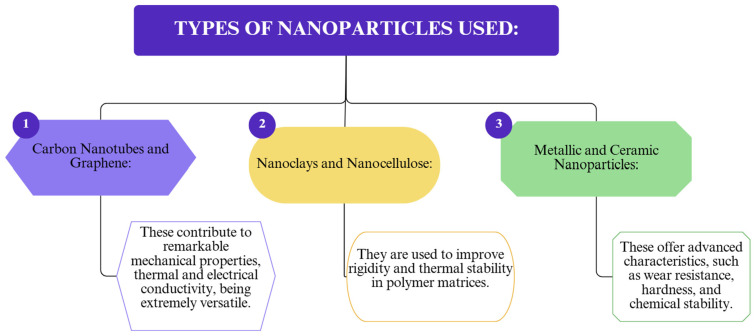
The main types of nanoparticles used.

**Figure 9:**
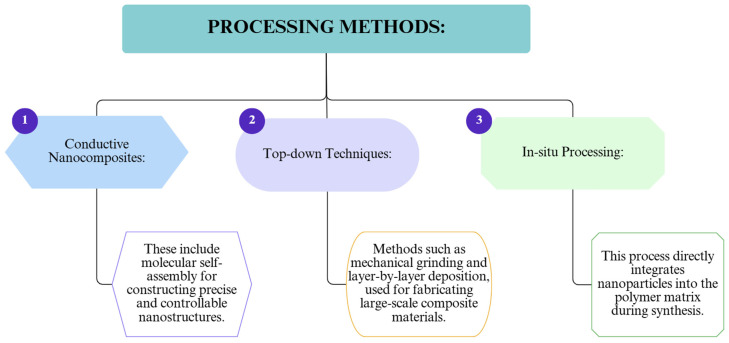
The main processing methods.

**Figure 10:**
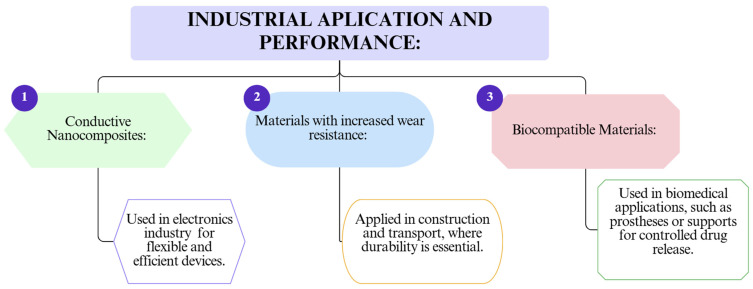
The main industrial applications and performance.

To illustrate the current advances in enhancing insulation materials via nanotechnology, key categories of nanoparticles, advanced processing methods, and major industrial applications were analyzed. Figure 8 displays the most commonly used nanoparticles in polymer composite formulations aimed at improving mechanical, thermal, or barrier properties. Figure 9 outlines the main processing techniques employed to integrate nanomaterials into polymer matrices, while Figure 10 highlights the industrial applications and performance improvements derived from nanostructuring strategies.

Through these analyses, the researchers [55] demonstrated that optimizing the interaction between polymers and nanoparticles by controlling nanoscale morphology is a key factor in improving material performance. They also emphasized that adopting advanced processing methods could enable the application of these materials in new technological fields, significantly impacting technological progress and applied research [55]. The article “Obtaining Nanostructured Surfaces: A Value Chain for the Use of Advanced Materials in Multisectoral Applications” explores the creation of nanostructured surfaces and their role in utilizing advanced materials for multisectoral applications. Researcher [56] highlights the importance of simulation and design methods in developing systems with controllable properties. A key aspect of the study is the use of nanomaterials to enhance surface properties such as wear resistance, corrosion resistance, selective adsorption, and biocompatibility. The production methods include wet chemical processes and physicochemical deposition in plasma or high vacuum environments, with a focus on the sol-gel technique for synthesizing nanometric powders [56].

Researchers [57] in the paper “Enhanced performance nanostructured thermoelectric converter for self-powering health sensors” demonstrated that semi-transparent perovskite solar cells have outstanding potential to overcome the traditional limitations of photovoltaic devices. In particular, replacing the conventional Spiro-OMeTAD material with a solution-processed hole transport layer (HTL) based on VNPB led to significant improvements. Solar cells constructed with this HTL achieved record performance values suitable for semi-transparent devices while maintaining an average visible transmittance between 10% and 30%. This research offers a promising alternative for the fabrication of efficient and durable semi-transparent solar cells, opening new perspectives for their integration into modern architecture and other industrial applications.

In conclusion, the most commonly used materials include epoxy resins, polyurethane, silicones, and fiberglass composites, each having different applications depending on their characteristics. Polymeric foams have proven effective for thermal insulation, offering excellent performance and a significant impact in improving energy efficiency. Recent research focuses on enhancing the performance of insulation materials by using nanomaterials, eco-friendly polymers, and advanced manufacturing technologies such as 3D printing.

**Q4. What future research is needed to improve the performance of insulation materials and develop innovative solutions?**

Future research should focus on advanced techniques that can significantly improve the performance of insulation materials, reduce their environmental impact, and extend their durability. In this regard, particularly promising research directions include the development of smart polymers, exploration of bioinspired materials, use of advanced computer simulations, and optimization of recycling processes.

- **Development of smart polymers**

Smart materials have the ability to respond to external factors or environmental changes by rearranging their molecular structure and adapting their functionality accordingly [58]. Phase-change polymeric materials have advanced significantly, integrating composites with optimized thermal conductivity and improved stability—critical aspects for energy efficiency. Their performance is enhanced through microencapsulation and integration of nanocomposites, expanding their applicability [59]. Moreover, the use of bio-based polymers supports the sustainability of these materials. PCMs are already used in electronics, textiles, and construction, as well as in medical therapies and packaging, where they help regulate temperature. It has also been shown that polymer-based PCMs can significantly contribute to global sustainability and the reduction of greenhouse gas emissions, positioning them as essential materials for the energy-efficient technologies of the future [59].

Other researchers [58] have used a multidisciplinary approach to analyze and demonstrate the mechanisms and properties of polymer-based smart materials.

A complex approach was employed for developing smart polymer materials based on controlled synthesis and advanced polymerization. Material analysis was carried out using spectroscopic methods to identify structural changes. Their behavior was examined under various external influences (temperature, electric/magnetic field), evaluating their resistance and flexibility. Ultimately, the materials were validated in real-world applications, testing their durability and adaptability in diverse environments.

Through this approach, it was demonstrated that smart materials can respond quickly and effectively to external stimuli, paving the way for various applications in technology and industry [58]. Recent advances in smart polymer gels have shown that these materials can modify their structure under the influence of external stimuli such as temperature, electric/magnetic fields, or chemicals.

Researchers [60] investigated the adaptability and resilience of these gels, highlighting their ability to respond rapidly to environmental changes. Applications target biomedicine, pharmaceutical engineering, and molecular devices, with high potential for controlled drug delivery and sensors. Future research directions include integrating multiple functionalities and increasing the durability of these materials [60].

- **Exploration of bioinspired materials**

Studying natural structures, which are already efficient in terms of thermal and structural performance, can lead to better insulation materials. Bioinspired principles such as porosity and multilayered structures can help develop more efficient and durable materials.

The article “Bioinspired Engineering of Thermal Materials” analyzes the use of ordered mesoporous nanofibers for advanced applications in lithium-metal batteries. Researchers [61] developed an innovative self-assembly method to create nanofibers with structures similar to vascular bundles found in nature. These structures facilitate efficient ion and electron transport, improving electrochemical performance.

In this research, the following were analyzed:

- Synthesis methods:

- Molecular self-assembly into ordered structures.
- Use of electrospinning to produce aligned nanofibers.

- Material characterization:

- Structural and functional analyses to evaluate porosity and conductivity.

- Battery performance:

- Testing of electrodes made from nanofibers in lithium-metal batteries.

Results demonstrated the following:

Ordered mesoporous nanofibers provide a larger active area and accelerated ion mobility, resulting in superior battery performance.

Nature-inspired structures can be used to improve the efficiency and durability of electrochemical devices [61].

Another thermally conductive bioinspired coating material based on cellulose nanofibers (CNFs) and boron nitride (BN) was developed using a co-elimination process and interfacial design to create a thermally efficient structure (13.8 W/m·K) with improved mechanical properties. The resulting material shows excellent adhesion and is considered highly suitable for industrial applications and electronic packaging. Due to its sustainability and ease of processing, this bioinspired material opens new perspectives in thermal management for modern devices [62].

- **Advanced computer simulations**

Advanced computer simulations play a crucial role in optimizing polymer resins by enabling the prediction of mechanical, thermal, and chemical properties at the molecular level.

Researchers [63] analyzed shape-memory polymers (SMPs) as smart materials capable of changing shape and returning to their original form under external stimuli. Figure 8 also explores activation mechanisms, polymer structure, and their applications in various fields.

Improving structural organization through the use of liquid crystalline elastomers enhances their responsiveness and durability. Due to their flexibility, SMPs have wide applications in biomedicine, flexible electronics, and advanced materials technology. Integrating composites and new functionalities such as conductivity or multistage memory opens innovative perspectives for industry and research [63].

**Figure 11:**
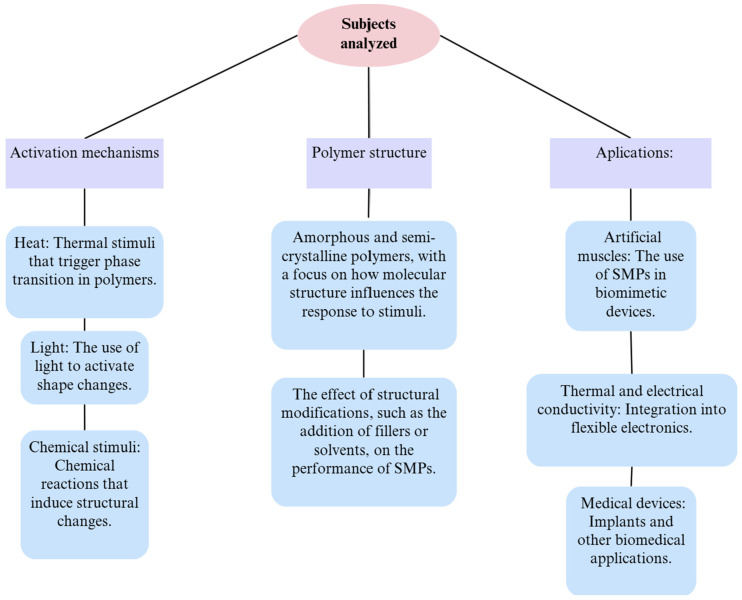
Main research topics identified in the literature regarding optimization and sustainability of polymeric insulation materials.

To provide a comprehensive overview of the research focus across the reviewed literature, a thematic classification of key topics was conducted. Figure 11 summarizes the main areas addressed in the selected studies, including smart polymers, advanced manufacturing technologies, bioinspired materials, composite recycling, and simulation-based optimization. This mapping facilitated the identification of research gaps and informed the future research directions proposed in this work.

Using an advanced SPACIER system that combines molecular simulations with machine learning to accelerate the design of innovative polymers—leveraging the RadonPy library and Bayesian optimization—researchers [64] demonstrated its ability to generate materials with optimized properties, such as advanced optical polymers. The platform reduces costs and experimental time, accelerating the development of innovative polymeric materials for multiple industrial applications [64].

## 5. Critical Discussion and Comparison of the Literature

The literature review reveals a wide variety of approaches to optimizing the performance of polymeric materials for insulation, each offering valuable contributions as well as specific limitations. Studies focused on epoxy resins, such as that by [6], highlight their advantages in terms of adhesion and chemical resistance. Other studies [18,22] have highlighted the challenges associated with recycling and thermal vulnerability, emphasizing the need for material reformulation or the integration of advanced additives to address these limitations.

In comparison, polymer foams analyzed by [37] offer an efficient alternative in terms of thermal performance and versatility, yet they are less durable under extreme conditions, requiring improvements in mechanical stability. Furthermore, graphene and carbon nanotubes, discussed in the works of [38,40], provide a significant leap in the thermal performance of composites, but pose challenges related to uniform dispersion in the polymer matrix and high production costs. Recent studies [47,48] exploring emerging technologies such as 3D printing and artificial intelligence integration in resin processing propose promising methods for customization and optimization. These technologies remain largely at the experimental or prototype stage and require additional validation in real-world industrial environments.

Regarding durability and sustainability, biodegradable polymers such as PHA or PLA are promoted in the literature [31,42] for their environmental compatibility. Still, their mechanical properties and stability must be improved for demanding industrial applications, as confirmed by studies on nanocomposites and hybrid materials.

In conclusion, the literature provides a wide range of solutions, yet none is universally optimal. The most promising results appear to emerge from combining emerging technologies (e.g., 3D printing, nanomaterials) with sustainable and adaptable polymers, paving the way for hybrid solutions that balance performance, durability, and ecological impact.

A comparative evaluation of polymeric systems, processing technologies, and application contexts reveals significant trade-offs that must be considered in designing insulation materials for automotive use.

- Material systems—Epoxy resins, widely studied for their mechanical strength and chemical stability, exhibit excellent adhesion and thermal resistance. Despite their advantageous properties, these materials exhibit poor recyclability and increased brittleness under thermal cycling, which restricts their applicability in demanding automotive conditions [6,22]. In contrast, polyurethane-based composites offer better flexibility and ease of processing, making them suitable for vibration-prone components, but they degrade under UV exposure and may emit toxic byproducts during thermal aging [33]. Silicone materials provide superior thermal stability and elasticity, yet their lower adhesion to substrates and high cost restrict their broader adoption [32].
- Processing methods—Traditional thermosetting approaches allow for high-performance curing but complicate end-of-life recycling. Additive manufacturing (e.g., DLP 3D printing) enables custom geometries and embedded functionality (such as smart sensors or self-healing layers), but scalability remains limited [47,48]. Nanostructuring, via incorporation of fillers such as graphene or CNTs, enhances performance across metrics but suffers from challenges in uniform dispersion, increased cost, and industrial reproducibility [40].
- Application contexts—Materials for passive components (e.g., housings, insulation panels) prioritize long-term thermal resistance and cost-efficiency, favoring polyester or fiberglass composites. In contrast, applications involving thermal shock or electromagnetic interference (e.g., battery enclosures or control units) benefit from hybrid aerogels or phase-change smart polymers that combine insulation, mechanical strength, and thermal management [51,59]. Automotive lightweighting requirements further complicate selection, as denser composites may offer better performance but at the expense of fuel efficiency.

By systematically comparing the technical, economic, and sustainability aspects of each solution, this review contributes to a more informed material selection strategy and highlights avenues for optimizing insulation materials tailored to the multifaceted demands of automotive applications.

Epoxy resins offer exceptional chemical resistance and structural stability, making them ideal for demanding applications. Although effective in many respects, their thermoset structure complicates recycling processes, and their susceptibility to thermal shock significantly limits their applicability in dynamic operating conditions. Compared to polyurethanes, which are more flexible and easier to process, epoxy systems offer better adhesion and thermal resistance, but at a higher ecological cost. Silicone resins surpass both in temperature resistance but exhibit weaker adhesion and higher production costs. These trade-offs are essential when selecting materials for thermal insulation in automotive contexts.

To support the comparative analysis presented above, Table 4 summarizes the key characteristics of four relevant polymer systems used in automotive thermal insulation.

In summary, the reviewed literature highlights a diverse range of polymeric systems and processing technologies for thermal insulation in automotive applications. However, no single solution proves universally optimal. Epoxy resins demonstrate high thermal resistance and strong adhesion but suffer from brittleness and low recyclability. Polyurethane systems offer flexibility and ease of processing but face limitations under UV exposure and may emit toxic byproducts during aging. Silicone-based materials excel in thermal stability and elasticity, though their high cost and limited adhesion constrain broader use. Meanwhile, fiberglass-reinforced composites and bio-based hybrid systems hold promise for passive insulation and sustainable solutions, respectively, yet each presents trade-offs in mechanical durability or industrial scalability.

Regarding manufacturing, conventional thermosetting techniques deliver reliable curing but hinder end-of-life recyclability. Emerging approaches such as digital light processing (DLP) and additive manufacturing enable customized geometries and the integration of smart functionalities (e.g., self-healing), though they remain limited to small-scale or experimental applications. Nanostructuring methods further enhance material performance, particularly thermal conductivity and strength, but are challenged by filler dispersion and production cost.

Application-specific requirements—such as resistance to thermal shock, lightweighting, or electromagnetic shielding—further complicate material selection. For instance, passive insulation panels may benefit from polyester or fiberglass systems, while high-performance battery enclosures or electronics modules require hybrid aerogels or phase-change smart polymers.

Therefore, the most promising solutions lie in the strategic combination of advanced technologies and sustainable materials tailored to specific performance needs. Hybrid systems that integrate bio-based matrices, nanofillers, and smart functionalities offer a viable path forward for automotive thermal insulation, balancing durability, processability, and environmental responsibility.

## 6. Conclusions

High-performance polymer resins have become a foundational pillar of modern industrial progress, being used to protect and optimally adapt industrial components. These materials represent a technological transformation, offering innovative solutions to increasingly complex production challenges. This study systematically explores the implications, examining their role in improving manufacturing processes and ensuring sustainability.

A primary consideration is the insulation of industrial components, which provides more than just protection—it is also a key element for the durability and efficiency of equipment. Proper application of insulation prevents material degradation due to external influences such as extreme temperature, humidity, or chemicals, thus extending the lifespan of products. Epoxy and polyurethane resins, for example, have become essential pillars in combating corrosion and energy losses.

While innovation drives progress, it also introduces challenges—particularly regarding the environmental footprint of synthetic materials. Consequently, the industry must reconsider manufacturing and recycling practices to mitigate its ecological impact. Solutions such as the use of biodegradable polymers and nanomaterials offer promising prospects. For example, incorporating graphene into the polymer matrix can significantly enhance thermal performance while maintaining sustainability.

Moreover, advanced technologies such as 3D printing and self-healing smart materials create opportunities for a better future where optimization means not only efficiency, but also adaptability and ecological responsibility. These technologies allow for the customization and repair of materials directly at the site of use, reducing costs and resource consumption.

The future research directions proposed in this article open significant opportunities for transforming the insulation materials industry. The creation of smart polymers proposes advanced solutions, such as self-healing materials and those adaptable to external stimuli, which can increase equipment efficiency and durability. At the same time, the study of bioinspired materials suggests using natural principles to develop multilayered and porous structures that are both thermally and structurally efficient. High-performance computer simulations enhance the research process by analyzing material behavior based on molecular structure, facilitating their optimization before manufacturing. These directions address challenges such as sustainability and recyclability, proposing solutions that reduce the ecological impact of synthetic materials. With such innovations, industrial insulation will no longer be just about utility but will become an essential element in global efforts for environmental protection and energy efficiency.

A critical comparison across material systems and application contexts reveals that hybrid and tailored solutions—rather than universal formulations—are essential for addressing the multifaceted demands of automotive thermal insulation. This review emphasizes the need for application-specific trade-offs and the strategic integration of sustainable, high-performance materials through advanced processing routes.

In conclusion, unlike previous studies, this research explores not only the conventional use of polymer resins but also the impact of new technologies on their sustainability and recyclability. As these materials adapt to modern requirements, the future of the industry can become more efficient, more durable, and more aware of its impact on the planet.

This systematic review lays the groundwork for targeted innovation in polymer insulation technologies, with the potential to drive substantial advancements in industrial performance and environmental sustainability.

Future research should explore hybrid materials that combine bio-based components with nanostructured fillers to balance performance and sustainability in thermally demanding automotive environments.

## Figures and Tables

**Table 1:** Synthesis of insulation materials.

Material	Key Properties	Advantages	Disadvantages
Epoxy resins	High mechanical strength, chemical stability, good adhesion	Excellent durability, high chemical resistance, versatile applications	High cost, may require additives or structural fillers to meet advanced thermal conditions
Polyurethane	Flexible, good thermal and acoustic insulation, moisture resistance	Easy to apply, high efficiency, water resistant	UV-sensitive, may release toxic substances over time
Polyesters	Moderate mechanical strength, decent thermal stability	Low cost, easy processing, multiple industrial uses	Brittle compared to other resins, sensitive to humidity
Silicones	High elasticity, resistance to extreme temperatures, hydrophobic	Long lifespan, weather resistant, highly flexible	High cost, limited adhesion to certain substrates
Acrylic resins	Good UV resistance, chemical stability, transparent	Yellowing resistant, lightweight, versatile in applications	Lower mechanical strength compared to epoxies or polyurethanes

**Table 2:** Summary Table of 20 Selected References.

Ref. No.	Aim of the Study	Material/System Studied	Key Findings	Application Area	Source
[1]	Review of composite materials in automotive applications	Various automotive composites	Summarizes benefits of composites in reducing weight and improving efficiency	Automotive	Khan et al. (2024)
[3]	Study on resin-based composite materials in insulation	Resin-based composites	Highlights advantages of electrical insulation and durability	Electrical/Automotive	Yan et al. (2023)
[5]	Overview of resin-based polymers	Epoxy resins	Demonstrates anti-corrosion properties and versatility	Coatings/Automotive	Umoren et al. (2022)
[6]	Review on epoxy resins as anticorrosive materials	Epoxy resins	Effective in protecting metals in harsh environments	Corrosion Protection	Verma et al. (2020)
[9]	Development of high thermally conductive polymer composites	All-polymer composites	Enhanced thermal conductivity for insulation	Thermal Management/Automotive	Ren et al. (2023)
[13]	Evaluation of insulation performance of anhydride-cured epoxy resin	Epoxy resin	Improved thermal insulation through curing regime control	Automotive/Electrical	Li et al. (2023)
[15]	Mechanical evaluation of carbon fiber-reinforced plastic	Carbon fiber-reinforced plastic	Analyzes tensile and bending loads	Automotive/Aerospace	Haldar et al. (2022)
[16]	Thermal degradation of epoxy-modified resins	Epoxy-modified resins	High temperature tolerance and multifunctional properties	Multifunctional Applications	Ciprioti et al. (2024)
[17]	Improving thermal conductivity in epoxy resin	Epoxy resin with rigid groups	Increased thermal conductivity for thermal shock conditions	Electronics/Automotive	Zhu et al. (2024)
[18]	Synthesis of new epoxy polymers	Epoxy polymers	Viscosimetric and rheological improvements	Thermal/Mechanical Insulation	El-Aouni et al. (2024)
[21]	Eco-design of polymer matrix composite parts	Eco-composites	Guidelines for sustainable material selection	Automotive/Green Design	Lazăr et al. (2023)
[22]	Recycling of anhydride-cured epoxy resin	Recycled epoxy resin	Describes degradation and reuse methods	Sustainable Manufacturing	Zhang et al. (2025)
[23]	Oxidative degradation for ammonia-cured epoxy recycling	Epoxy resin	Use of nitric acid to enable material recovery	Circular Economy	Zhang et al. (2023)

**Table 3:** The most used materials for insulating components.

Material	Property and Application
Epoxy resins	These have a strong adhesion capability to various substrates, especially metal surfaces, with low shrinkage—unlike most thermosetting polymer-based adhesives, which are associated with low-resistance polymers [6]. Obtained through a chemical condensation reaction of ethylene oxides (epoxides) with polyphenols, diols, or amines, they are used as strong adhesives for various materials (concrete, metal, glass, wood, etc.), in industrial flooring, concrete element repair, and protective coatings [32].
Polyurethanes	Obtained through a chemical condensation reaction between diisocyanates and alcohols, they are used as flexible foams for upholstery and rigid ones for thermal and acoustic insulation [32]. They combine the hardness and rigidity of metals and plastics with the flexibility of rubbers. This preferable point of material properties and characteristics has made polyurethane the material of choice for many applications, even over metals and plastics [33].
Polyetheretherketone (PEEK)	It is a thermoplastic polyaromatic polymer that exhibits high thermal and chemical stability. It is also a high-performance semicrystalline polymer produced using stepwise polymerization through double alkylation of bisphenol salts [34].
Silicones	They are obtained through the polycondensation of siliceous acids with certain organic substances; used as liquid silicones and as lubricants, as well as in the production of fairly durable paints; silicone rubber is used for sealing gaskets that work in variable climates [32].
Materials: fiberglass composites	Fiberglass is a material made up of multiple extremely fine glass fibers, and it has mechanical properties comparable to other fibers such as polymers and carbon fiber [35]. Recently, more research has been conducted on fiberglass-reinforced composites due to their excellent mechanical properties [36].

**Table 4:** Comparative Overview of Polymer Systems for Automotive Thermal Insulation.

Polymer System	Thermal Resistance	Recyclability	Processing Ease	Typical Application	Key Limitation
Epoxy Resins	High	Low	Moderate	Battery enclosures	Brittle, non-recyclable
Polyurethanes	Moderate	Moderate	High	Interior insulation	UV degradation, toxicity
Silicones	Very High	Low	Low	Seals, electronics	High cost, weak adhesion
Bio-based Hybrids	Moderate–High	High	Developing	Sustainable insulation panels	Limited industrial testing
Fiberglass Composites	Moderate	Low	High	Housings, body panels	Brittle under vibration

## Data Availability

The original contributions presented in this study are included in the article. Further inquiries can be directed to the corresponding author.

## Supplementary Materials

The following supporting information can be downloaded at https://www.mdpi.com/article/10.3390/polym17162200/s1, PRISMA 2020 Checklist.

## Abbreviations

BN	Boron Nitride
CNFs	Cellulose Nanofibers
CO_2_	Carbon Dioxide
CNT	Carbon Nanotubes
DLP	Digital Light Processing
HCP	Hexagonal Close-Packing
HOI	Inorganic Oxide Heterostructures
HTL	Hole Transport Layer
OMeTAD	O-methyl-2,2′,7,7′-tetramethyl-spiro[fluorene-9,9′-xanthene]-2,2′-diol
PCM	Phase-Change Materials
PEEK	PolyEtherEtherKetone
PHA	Polyhydroxyalkanoate
PLA	Polylactic Acid
PRISMA	Preferred Reporting Items for Systematic Reviews and Meta-Analyses
RAFT	Reversible Addition–Fragmentation Chain Transfer
SMPs	Shape-Memory Polymers
SPACIER	Smart Polymer Artificial Chemical Intelligence Engine for Research
VNPB	N,N’-Bis(naphthalen-1-yl)-N,N’-bis(phenyl)-benzidine
W/m·K	Watts per meter-kelvin
