# Supplementary material for: Sustainable Polymer Composites for Thermal Insulation in Automotive Applications: A Systematic Literature Review
Source: Polymers (Basel). 2025 Aug 12;17(16):2200. doi: 10.3390/polym17162200 (PMC12389532; doi:10.3390/polym17162200)
Supplement: Supplementary file 1 [file polymers-17-02200-s001.zip › polymers-3765430-supplementary.pdf]

PRISMA 2020 Checklist

This checklist was completed in accordance with the PRISMA 2020 guidelines, available at https://www.prisma-statement.org/. It corresponds to the systematic review titled "Sustainable Polymer Composites for Thermal Insulation in Automotive Applications: A Systematic Literature Review".

Section and Topic	Item #	Checklist Item	Location where item is reported
TITLE	1	Identify the report as a systematic review.	Title page
ABSTRACT	2	Provide a structured summary including background, objectives, data sources, study eligibility criteria, participants and interventions, study appraisal and synthesis methods, results, limitations, conclusions, and registration information.	Abstract
INTRODUCTION	3	Describe the rationale for the review in the context of existing knowledge.	Introduction, paragraph 1
INTRODUCTION	4	Provide an explicit statement of the objective(s) or question(s) the review addresses.	Introduction, final paragraph
METHODS	5	Specify the inclusion and exclusion criteria for the	Methods, section 2.1

		review and how studies were grouped for the syntheses.	
METHODS	6	Specify all information sources (e.g., databases with dates of coverage) and the date when each was last searched.	Methods, section 2.2
METHODS	7	Present the full search strategies for all databases, registers and websites, including any filters and limits used.	Supplementary Table 1
METHODS	8	Specify the methods used to decide whether a study met the inclusion criteria.	Methods, section 2.3
METHODS	9	Specify the methods used to collect data from reports.	Methods, section 2.3
METHODS	10	List and define all outcomes for which data were sought and methods for handling and combining results.	Methods, section 2.4
METHODS	11	Describe methods used to assess risk of bias in the included studies.	Methods, section 2.5
METHODS	12	Specify methods for the synthesis of	Methods, section 2.6

		results and rationale for the choices.	
METHODS	13	Describe any methods used to explore heterogeneity among study results.	Methods, section 2.6
METHODS	14	Describe any sensitivity analyses conducted to assess robustness of results.	Methods, section 2.6
RESULTS	15	Provide a flow diagram and information on the number of studies screened, assessed for eligibility, and included.	Figure 3 (PRISMA flow diagram)
RESULTS	16	For each included study, present characteristics for which data were extracted.	Results, section 3.1
RESULTS	17	Present data on risk of bias for each study.	Results, section 3.1
RESULTS	18	Present results of individual studies and syntheses.	Results, section 3.2–3.4
RESULTS	19	Present results of investigations into possible causes of heterogeneity.	Results, section 3.4
RESULTS	20	Present results of sensitivity analyses.	Results, section 3.4

DISCUSSION	21	Provide a general interpretation of the results in the context of other evidence.	Discussion, paragraph 1
DISCUSSION	22	Discuss limitations of the evidence included in the review.	Discussion, paragraph 3
DISCUSSION	23	Discuss limitations of the review processes used.	Discussion, paragraph 4
DISCUSSION	24	Discuss implications of the results for practice, policy, and future research.	Discussion, final paragraph
OTHER	25	Describe sources of funding and other support and the role of funders.	Funding, Acknowledgements
OTHER	26	Report any competing interests of review authors.	Conflicts of Interest
OTHER	27	Provide registration information for the review and availability of the protocol.	Methods, section 2.1
